# Genetic Diversity and Population Structure of Sika Deer (*Cervus nippon*) Inferred by mtDNA and Y-Chromosomal Genes

**DOI:** 10.3390/ani15203022

**Published:** 2025-10-17

**Authors:** Tianjiao Wang, Yimeng Dong, Lei Wang, Huamiao Liu, Weilin Su, Xiumei Xing

**Affiliations:** State Key Laboratory for Molecular Biology of Special Economic Animals, Institute of Special Animal and Plant Sciences, Chinese Academy of Agricultural Sciences, Changchun 130112, China; wangtianjiao@caas.cn (T.W.); dongyimeng1018@126.com (Y.D.); liuhuamiao2000@163.com (H.L.);

**Keywords:** sika deer, mitochondrial genome, Y chromosome, maternal origin, paternal origin, tandem repeat unit

## Abstract

**Simple Summary:**

A systematic investigation of the genetic diversity and structure of different sika deer populations is conducive to their population management, protection, and reproduction. Using mitochondria and genes on the Y chromosome, we found that the sika deer population consists of nine maternal lineages and three paternal lineages. The overall maternal genetic diversity of sika deer is relatively high, while that of the paternal line is low. In addition, the tandem repeat units in the control region in Japanese populations exhibit significantly higher diversity in both type and copy number. Notably, we identified a 26 bp tandem repeat motif unique to southern Japanese populations.

**Abstract:**

Sika deer (*Cervus nippon*), a species mainly distributed in the northeast of Asia, hold significant economic value in China due to their contributions to traditional Chinese medicine. A systematic investigation of their genetic structure is needed for population management. In this study, mitochondrial genome and AMELY, DBY, USP9Y, and SRY gene fragments on Y chromosome were used to elucidate the genetic structure of 303 individuals across 8 distinct populations. The mitosome analysis identified 72 haplotypes, with a haplotype diversity (Hd) of 0.917 and nucleotide diversity (π) of 0.0143, respectively. Meanwhile, 13 haplotypes were defined by Y chromosome genes with a Hd of 0.791. Analysis of the mitochondrial control region (CR) revealed subspecies-specific patterns of tandem repeat unit organization between continental and Japanese groups. Y chromosome analyses demonstrated a homogeneous paternal lineage across Japanese populations.

## 1. Introduction

Sika deer (*Cervus nippon*), classified under the Artiodactyla, represents a key species in the East Asian monsoon region. Their distribution spans from the Wusuli River to Vietnam [1,2]. Known for their significant economic value, the wild population of sika deer in China has drastically declined due to habitat destruction and environmental degradation, rendering them an endangered species [3,4,5]. Based on morphological characteristics, the sika deer is currently categorized into 13 subspecies, including six in China, six in Japan, and one in Vietnam [6]. Recent research suggests that Chinese and Japanese sika deer may be considered as distinct phylogenetic species [6]. In Japan, the sika deer population has grown substantially, exceeding three million individuals. This overpopulation, attributed to their herbivorous diet, has led to severe vegetation damage, surpassing the ecological carrying capacity of their habitats. Additionally, frequent appearances near roads and railways have resulted in numerous traffic accidents, disrupting the daily lives of local residents [7,8]. In China, sika deer breeding dates back over 300 years ago, with the current domesticated population primarily descending from the Northeast subspecies [9]. Through selective breeding, seven genetically distinct varieties have been developed.

Mitochondrial DNA (mtDNA) strictly follow the characteristics of maternal unisexual inheritance. A single sample can carry the entire maternal lineage signature of a population, representing the characteristics of a maternal group. This allows for a comprehensive analysis of a population’s genetic structure with relatively few samples, yielding reliable results which are highly beneficial for population genetic studies. Genes such as cytochrome b (CYTB), the control region (D-loop), 12S rRNA, and 16S rRNA are common hotspots in mtDNA research. Tamate H.B. et al. sequenced a 367 bp segment of the CYTB gene across seven subspecies (*C. n. kopschi*, *C. n. yesoensis*, *C. n. centralis*, *C. n. nippon*, *C. n. mageshimae*, *C. n. yakushimae*, and *C. n. keramae*) and discovered nine haplotypes. The analysis revealed that the dividing line between the north and south groups of Japanese sika deer lies on Honshu Island, not in the straits between islands, and suggested the Ryukyu subspecies might be the descendants of deer originally introduced from Kyushu Island [10]. Lyu X.P. et al. sequenced a 335 bp segment of the D-loop region from 45 individuals across four populations (*C. n. hortulorum*, *C. n. sichuanicus*, *C. n. kopschi*, and *C. n. taiouanus*). The analysis identified eight haplotypes. Phylogenetic relationships indicated that the samples are genetically closer to southern Japanese deer than to northern Japanese deer [11]. Wu et al. found that four populations exhibited high gene flow but low genetic diversity. Based on mtDNA variation, they identified two major phylogenetic clades [12].

There may be some patterns in the tandem repetitive sequences of the D-loop regions in the mitochondria of sika deer. Nagata J. et al. noted differences in the number of tandem repeat units between the two major Japanese lineages: 6–7 in northern population, 4–5 in southern population, and, meanwhile, 4 in East Asian continent. The first repeat unit was identified as ancestral, with subsequent units formed via replication slippage. Thus, the number of repeat units serves as a genetic marker for distinguishing populations [13,14]. In addition, Ba et al. extracted and analyzed 1023 tandem repeat units from 243 D-loop sequences and identified 52 haplotypes which classified the repeat units into three groups, and the mechanism of the increase in the number of tandem repeat units was explored [15].

During generational transmission, the Y chromosome is passed only from the male parent to the male offspring. Consequently, the Y chromosome serves as an effective molecular marker for studying paternal lineage genetics and evolution. As a haploid chromosome, the Y chromosome exhibits a higher frequency of insertion/deletion (indel) mutations compared with autosomes, and has a smaller effective population size. This makes it highly significant when studying species genetic diversity. Research found that contrary to mtDNA patterns, there was no clear differentiation of Y chromosome markers between the southern and the northern Japanese populations [16]. Research on the Y chromosome mainly focuses on several protein encoding genes, such as amelogenin Y-linked (AMELY), sex-determining region Y (SRY), DEAD-box helicase 3 Y-linked (DBY), and Zinc finger protein Y-linked (ZFY) [17,18,19].

In this study, we sequenced the mitochondrial genomes and AMELY, DBY, USP9Y. and SRY genes on the Y chromosomes in 235 continental and 68 Japanese sika deer, respectively. The genetic diversity and evolutionary origin of sika deer were explored from both maternal and paternal perspectives, respectively. The findings are anticipated to offer valuable insights for subspecies classification, genetic background assessment, and the phylogenetic analysis of sika deer.

## 2. Materials and Methods

### 2.1. Sample Collection and DNA Extraction

**Figure 1:**
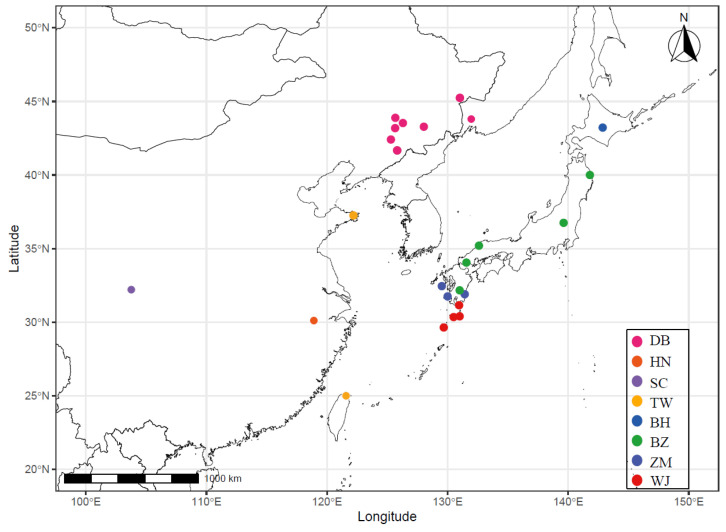
Locations of the sampled *Cervus nippon* populations. Dots of the same color represent different sampling sites of the same subspecies. Geospatial data from Natural Earth (https://www.naturalearthdata.com) was used for base mapping.

A total of 303 sika deer specimens (DNA, blood, tissues, and feces) were collected from four continental subspecies (*n* = 235) and four Japanese subspecies (*n* = 68) of sika deer (Figure 1, Table 1). Nucleotide sequences from 287 of the 303 individuals have already been reported by Dong et al. [20], and the remaining animals (16 samples) were newly analyzed in this study. Among all the samples, mitochondrial DNA sequencing was performed on 290 samples, while Y chromosome sequencing was conducted exclusively on 150 male specimens (Table S1).

Total DNA was extracted from blood and feces using Bioteke whole blood genomic DNA extraction kits (Bioteke Biotechnology Co., Ltd., Beijing, China) and QIAamp DNA Stool Mini kits (QIAGEN Bioengineering Co., Ltd., Shenzhen, China), respectively, following the manufacturer’s instructions. Phenol-chloroform method (1:1) was used to extract DNA from tissue samples. Extract without any tissue was used as a negative control in the subsequent polymerase chain reaction (PCR) amplification.

### 2.2. Primer Design and Synthesis

Primer Premier 5.0 and Oligo 6.0 were used to design primers covering the entire mitochondrial sequence and Y chromosome genes of sika deer, with fragment lengths of approximately 1.5 kb. The reference mitochondrial genome was HQ191428.1, published in NCBI database. The newly synthesized primers were then stored at −20 °C. Detailed information on primers is listed in Table S2.

### 2.3. PCR Amplification and Sequencing

The PCR amplification reaction mix contained 0.5 μL each of the upstream and downstream primers, 11.5 μL 2 × Es Taq MasterMix, 1 μL DNA template, and 11.5 μL ddH_2_O, totaling 25 μL. The PCR amplification was conducted under the following conditions: an initial denaturation at 94 °C for 5 min, followed by 30 cycles of amplification (94 °C for 30 s, 56 °C for 30 s, 72 °C for 70 s), and a final extension at 72 °C for 5 min. The PCR products were verified using 1% agarose gel electrophoresis, followed by staining with TS-GelRed Ver.2 10,000 × in Water (Tsingke Biological Technology Co., Ltd., Beijing, China). Products were sequenced at Tsingke Biological Technology Co., Ltd., Beijing, China.

### 2.4. Data Analysis

Sequences were assembled using DNAMAN v6 [21]. The bidirectional sequencing results are, respectively, imported into different channels. By default, the progressive alignment algorithm is used to generate consistent sequences. MEGA 12.0.11 was used to conduct multiple sequence alignment, using the MUSCLE algorithm with default parameters and calculating genetic distances between populations using the “Tajima-Nei” model and 1000 bootstrap [22]. The software (v12.0.11) was also used to construct maximum likelihood (ML) phylogenetic trees with the “K2P” model. The 13 protein-coding genes (PCGs) were concatenated using SequenceMatrix v1.78 software [23]. Haplotypes were defined using DnaSP v6.12.03, and haplotype diversity (Hd) and nucleotide diversity (π) were calculated [24]. Haplotype network diagrams were constructed using PopArt-1.7 with the “T-Coffee Sequence (TCS) network” method [25,26]. The Arlequin_v3522 software was employed to calculate interpopulation FST values and perform AMOVA analysis [27]. The TRFv4.09software was used to identify tandem repeats sequences in the control region (CR) of mitosomes, with the following parameters: minimum repetitive unit length = 2, minimum repetition times = 7, maximum allowable interval length = 7, percentage of mismatch = 80, minimum repetitive length = 10, maximum repetitive length = 50, and maximum sequence length = 2000 [28]. We manually aligned the other samples with the highest frequency of repetitive sequence patterns as a reference. The multiple sequence alignment file was imported into the ‘adegenet’ package in R v4.2.2 for discriminant analysis of principal components (DAPC) analysis [29,30]. The outgroup of the mitochondrial phylogenetic tree is derived from the homologous sequence NC_006853.1 of cattle in NCBI. During the phylogenetic construction of the Y chromosome, homologous sequences from the full-length Y chromosome of cattle (AC216937.5) were employed as the rooting reference.

## 3. Results

### 3.1. Different Patterns of Tandem Repeat Sequences

**Figure 2:**
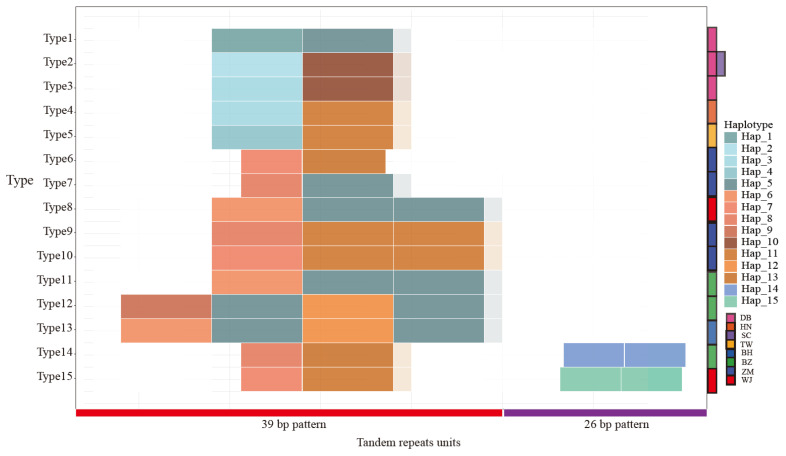
Haplotypes of repetitive tandem patterns in all 290 individuals.

The sequence of mitochondrial control regions in sika deer is located between tRNA (Phe) and tRNA (Pro), with a length ranging from 989 bp to 1100 bp. The results of the TR sequences revealed that a pattern of 39 bp repetitive sequences existed in all individuals. Additionally, the continental sika deer had only this kind of repetitive sequence with 2.2 copy numbers. However, the repetitive sequence patterns in the Japanese individuals were more numerous than those in the continental samples. This 39 bp pattern showed additional copies or omissions in the Japanese individuals. Moreover, in the southern BZ populations and certain individuals of WJ, repetitive sequences of a 26 bp consensus sequence were found at the distal end (Figure 2 and Tables S3 and S4). Among the total of 49 individuals from southern Japan, 21 were revealed to have the 26 bp repetitive pattern by TRF software, accounting for approximately 43%.

We conducted haplotype analysis on the consensus sequences of two repetitive units. The first tandem repetitive sequence (TR1) identified 13 haplotypes in consensus, and the second repetitive sequence (TR2) identified 2 haplotypes in consensus. A total of 15 permutation types were discovered. Except for the arrangement type of SC, which is the same as that of some individuals from DB, the other subspecies all have their own unique arrangement types. Types 1~5 are exclusive types of continental and Types 6~15 are types particular to Japan. Among them, types 11, 12, and 13 are unique to northern populations and the remaining belong to southern populations. The results indicate that the tandem repetitive sequences in the mitochondrial control region show more abundant structural diversity than in the Japanese population. In terms of the number of duplicate units, the Hokkaido subspecies and some BZ samples, namely the population in northern Japan, have the largest number of copies. Most southern Japanese samples have a small section missing in the repetitive units shared by the continental and Japanese populations. Additionally, the 26 bp repetition pattern is unique to the southern Japanese population.

### 3.2. Genetic Diversity of Mitochondrial Protein-Coding Genes

A total of 72 haplotypes were identified from 290 individuals based on 13 protein-coding genes (PCGs). The haplotype diversity (Hd) varied from 0.4 (±0.106), in the BH population, to 1 (±0.089), in the TW population, with an overall haplotype diversity of 0.917 (±0.000). Nucleotide diversity (π) ranged from 0.00014 (±3.58 × 10^−5^), in the BH population, to 0.0108 (±0.0004), in the BZ population, with an overall nucleotide diversity of 0.0143 (±4.58 × 10^−5^). The number of haplotypes per population ranged from two to ten, with no shared haplotypes among different subspecies. The BH population exhibited the fewest haplotypes (h = 2) and the ZM population displayed the most (h = 10), suggesting that the ZM population possesses the highest genetic diversity, whereas the BH population exhibits the lowest genetic diversity (Table 2 and Table S5).

**Figure 3:**
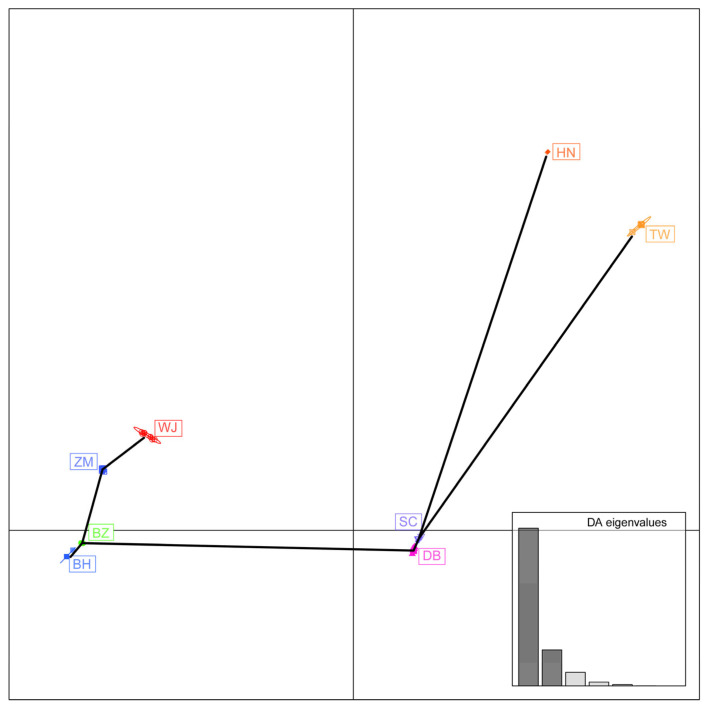
The distribution of eight sika deer populations by DAPC analysis.

A total of 916 SNPs were found in 13 protein-coding genes from all wild sika deer individuals. The number of subspecies-specific SNPs was 124 (Table 3). The subspecies-specific SNPs ranged from 1 in the DB population to 40 in the TW population. The DAPC analysis revealed that the Japanese population has significant genetic similarities, forming relatively independent genetic clusters; HN and TW are separated from other groups (Figure 3). The genetic distance among the eight populations varied from 0.0087 (DB/HN) to 0.0338 (WJ/TW), while the pairwise Fst values ranged from 0.053 (SC/TW) to 0.403 (BH/HN) (Table 4). Among those, the BH population exhibited significant genetic differentiation from all other populations (Fst > 0.25) (Figure S1). The AMOVA analysis of PCGs indicated that 84.51% of the genetic variation is attributable to differences within populations, whereas 15.49% occurred among populations (Table S6).

### 3.3. Genetic Diversity of Y-Chromosome Genes

**Figure 4:**
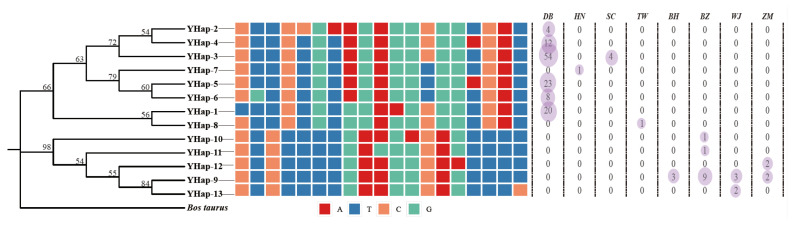
Phylogenetic trees and haplotypes distribution based on Y chromosome genes.

Four Y chromosome genes (AMELY, DBY, USP9Y, and SRY) were sequenced and linearly linked, with an aligned length of 4934 bp. A total of 19 polymorphic loci combined into 13 haplotypes were determined in all the 150 male individuals. Details of the haplotypes’ distribution are shown in Figure 4. Eight haplotypes were distributed in the continental population, while five haplotypes were presented in the Japanese population. No types were shared among them. In the haplotype phylogram, we found two divergent genetic lineages, the continental lineage and the Japanese lineage. The continental lineage included two clades, while the Japanese population were classified into one single clades.

**Figure 5:**
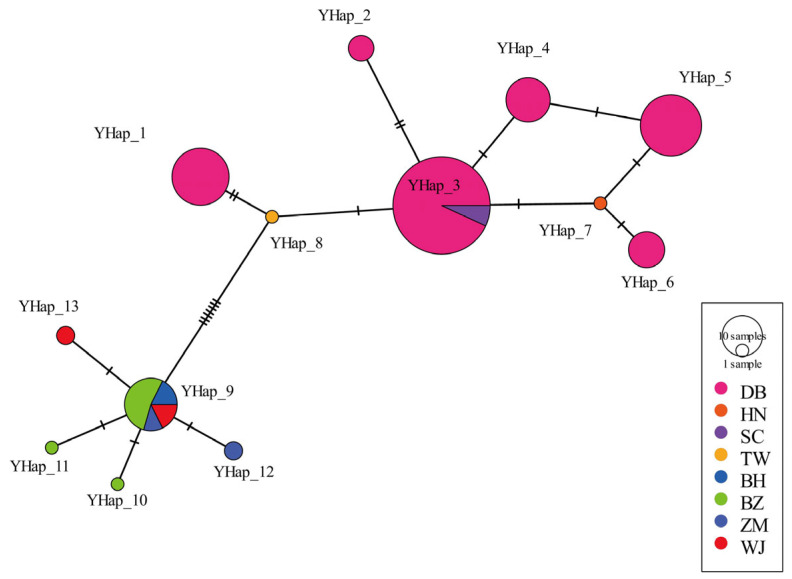
Haplotype network diagram of eight sika deer populations, based on Y chromosome genes.

Network analysis illustrated the frequency and distribution of Y chromosome haplotypes (Figure 5). Haplotype 3 proved to be the most dominant in the continental population, with a frequency 36%, appearing in both the DB and SC populations, and may serve as the most primitive haplotype. Haplotype 9 emerged as the most prevalent in the Japanese population, appearing in all four subspecies, and its frequency is 11%. The Japanese population may thus differentiated into new haplotypes. The BZ population exhibited three distinct haplotypes.

Similarly, the Y chromosome sequences were used to calculate the genetic distance between populations and population diversity. The Hd varied from 0.35, in the BZ population, to 0.73, in the DB population, excluding populations that have only one single haplotype. Nucleotide diversity also varied from 0.00007 to 0.00038 in these two populations. The overall Hd and π were 0.793 and 0.00075, respectively. The genetic distance between the DB and ZM population is the greatest, while the closest genetic distance occurs between the BH and WJ population (Table 5).

## 4. Discussion

This study obtained the complete mitochondrial genome sequences of 303 individuals from eight sika deer (*Cervus nippon*) subspecies: the Northeast subspecies (*C. n. hortulorum*), the Sichuan subspecies (*C. n. sichuanicus*), the South China subspecies (*C. n. kopschi*), the Taiwan subspecies (*C. n. taiouanus*), the Hokkaido subspecies (*C. n. yesoensis*), the Honshu subspecies (*C. n. centralis*), the Kyushu subspecies (*C. n. nippon*), and the Yakushima subspecies (*C. n. yakushimae*). Samples from the remaining three wild subspecies (the Matsushima, Ryukyu, and Vietnamese subspecies) were not collected, and no complete mitochondrial genome data for these subspecies were available in public genomic databases. Therefore, this study focuses on analyzing the mitochondrial genes and genes on the Y chromosome among these eight wild subspecies and domesticated populations.

In recent years, many studies have analyzed the genetic diversity of sika deer by means of mitochondrial genomes and microsatellites. Štohlová et al. analyzed the genetic diversity of sika deer populations, based on 11 microsatellite loci, demonstrating that sika deer possess relatively high genetic diversity [31]. Research findings indicated that the genetic diversity of sika deer in Northeast China is the highest, while that of Japanese sika deer is the lowest [32]. Zhou reported that the haplotype diversity (Hd) and nucleotide diversity (π) of male domestic sika deer, based on 13 protein-coding genes, are 0.8997 and 0.00477, respectively [33]. In the present study, the Hd and π of sika deer, based on 13 protein-coding genes, are 0.917 and 0.01430, respectively. Both values are at a relatively high level (Hd > 0.5, π > 0.005), suggesting that the overall maternal genetic diversity of sika deer is relatively high. In a previous work, Borowski Z. et al. calculated the Hd of the mitochondrial control region, using 357 red deer individuals from Poland, and found the overall Hd to be 0.90, suggesting that the pattern of maternal genetic diversity may also exist in other deer species [34]. The high level of genetic diversity, coupled with a low proportion of shared haplotypes among populations, may potentially suggest temporal genetic drift effects. The Taiwan subspecies exhibits the highest haplotype diversity. There are, in total, four individuals, with each individual representing a distinct haplotype. It is advisable to maintain their current management state. In the future, a further assessment of their genetic diversity can be conducted if it is possible to increase the sample size. The haplotype diversity and nucleotide diversity of the Hokkaido subspecies are the lowest. This may be associated with the bottleneck period that the Hokkaido subspecies has experienced, resulting in a low level of population genetic diversity [15,35,36]. However, it is worth noting that the number of subspecies from Hokkaido used in this study is limited, and there may be a bias in the estimation of diversity due to the insufficient sample size. Additionally, the study exhibits a significant disparity in the sample size between domestic and wild populations, with domestic samples being predominant. This imbalance may introduce methodological biases, as domesticated populations are subject to artificial selection pressures and controlled reproductive strategies, potentially amplifying minor genetic variations that could distort the overall assessment of genetic diversity within the dataset.

The total number of subspecies-specific SNPs is 124, which can serve as candidate molecular markers for distinguishing the Northeast, Sichuan, South China, Taiwan, Hokkaido, and Yakushima subspecies. No subspecies-specific SNPs were identified in the Honshu and the Kyushu subspecies, which may be attributed to the significant sequence divergence between the northern and southern populations of the Honshu subspecies. The northern Honshu population shows a higher sequence similarity to the Hokkaido subspecies, while the southern Honshu population exhibits a closer affinity to the Kyushu subspecies. Among the 13 protein-coding genes, NAD2 and NAD4L did not yield subspecies-specific SNPs and are therefore not recommended for subspecies discrimination.

In the control region (CR), the tandem repeat units (TRs) in the Japanese populations exhibit significantly higher diversity in both types and copy numbers compared to other groups. Inconsistent with prior research findings, the analysis of TR reveals that not all the northern Japanese populations have a higher copy number of TR than the southern Japanese populations. Notably, we identified a 26 bp tandem repeat motif unique to the southern Japanese populations, which has not been reported in previous studies [13,14,15]. In contrast, the continental populations demonstrated greater conservation in TR units. By analyzing the nucleotide variation sites, copy numbers, and arrangement patterns of these repeats, TRUs can serve as robust molecular markers for subspecies discrimination.

Based on the haplotype network and phylogenetic tree constructed from the 13 protein-coding genes (pcgs) of the mitochondrial genome of sika deer, this study divided sika deer into the continental group and the Japanese group, and further into four major clades and nine maternal lineages (Figure S2). This is consistent with the previous analysis, based on the complete mitochondrial genome [20]. More specifically, the DB population has the largest number of four maternal lineages, with two maternal lineages each in BZ and ZM, and one maternal lineage in all other subspecies. There are three maternal types of domestic sika deer, which indicates that the genetic background of domestic populations is not uniform. The domestic population was developed on the basis of the DB subspecies through years of artificial breeding. Compared with wild sika deer, its production performance has been significantly improved. At present, in the breeding of sika deer, a scientific mating system should be formulated based on the genetic background and kinship of sika deer. It is necessary to determine whether the maternal lineage is the original Northeast subspecies, or the type that has undergone gene flow with other subspecies. While maintaining the maternal diversity of the DB population, it is also very important to ensure the uniqueness of its bloodline, which distinguishes it from other subspecies. The Y phylogenetic tree shows that haplotypes H2, 3, 4, 5, 6, and 7 form monophyletic group I, haplotypes H1 and H8 form monophyletic group II, and haplotypes H9, 10, 11, 12, and 13 form monophyletic group III. This is consistent with the structure of the Y gene haplotype network, indicating that there may be three paternal lineages in this population of sika deer. Monophyletic groups I and II constitute the continental group, and monophyletic group III constitutes the Japanese group. For continental populations, only the DB population has two paternal lineages. All other groups have only one paternal lineage, while all Japanese populations share only one paternal lineage. The Y chromosome haplotype network shows that haplotype H3 is the dominant haplotype, shared by the Sichuan and Northeast subspecies, indicating that there is gene flow between the Sichuan and Northeast subspecies. To mitigate the genetic drift caused by the dominant haplotype, breeding programs should prioritize controlled mating among sika deer with low-frequency haplotypes. This approach not only sustains allelic diversity, but also enhances adaptive potential through optimized gene pool management.

Haplotype H9 is shared by the four subspecies of Japanese sika deer and has a frequency of 69.57% in Japanese sika deer, making it the dominant haplotype, and the only haplotype, of the Hokkaido subspecies. The results indicate that Japanese sika deer originated from a common paternal ancestral population, with H9 being the most primitive haplotype of Japanese sika deer, and other haplotypes evolving from H9 through base substitutions. It is worth noting that the analysis of Y chromosome haplotypes may be affected by the small number of samples from certain populations, such as South China and TW, included in the study. The polymorphism information content (PIC) per locus was calculated, and the PIC was found to range from 0.01 to 0.41 for 19 polymorphic sites. It must be admitted that low informative sites might compromise the resolution of demographic inference and population structure assessment.

The overall haplotype diversity and nucleotide diversity of sika deer, based on four Y chromosome gene fragments, were 0.793 and 0.00075, respectively. The nucleotide diversity of all subspecies was at a relatively low level. This may be due to the short length of the Y chromosome gene fragments and the few variation sites, on the one hand, and, on the other hand, it might be attributed to the polygamous mating system of sika deer. Moreover, the Y chromosome undergoes paternal inheritance and lacks recombination. This characteristic may cause an overestimation of population differentiation in terms of the Fst. Research has indicated that the Fst between populations estimated from the Y chromosome is significantly higher than that from autosomes [37,38]. Consequently, in this study, Y chromosome gene segments were not employed to calculate the Fst between populations.

A total of 137 specimens were tested for both mitochondria and Y chromosomes. The haplotype distribution is shown in Figure S3. It can be seen that the maternal haplotypes in the same group are more abundant than the paternal haplotypes. Different maternal haplotypes can share the same paternal haplotype. The sharing of the Y chromosome haplotypes reflects the selection pressure and the mating system of patrilineal inheritance, while the discrimination of mitochondrial haplotypes demonstrates the stability and the diversity of matrilineal inheritance. To maintain the genetic diversity of sika deer during artificial breeding, it is recommended to implement controlled mating pairings that exclude individuals sharing identical paternal lineages. In addition, the strategic introduction of individuals with distant maternal relationships can be implemented to promote gene flow. This strategy effectively mitigates the risks of inbreeding depression while preserving the heterozygosity of the population.

## 5. Conclusions

In this study, the genetic background of both East Asian continent and Japanese sika deer populations was analyzed using mtDNA and Y chromosome genes, providing a comprehensive examination of the genetic diversity and structure across different populations. Our findings indicate that the maternal genetic diversity of sika deer is at a relatively high level, while the paternal genetic diversity is relatively low. Different groups of sika deer have unique tandem repetitive sequence patterns in the control region, which may be used for the identification of different sika deer populations. Phylogenetic analysis of the Y chromosome reveals a monophyletic origin of the Japanese sika deer population, indicating shared paternal ancestry.

## Figures and Tables

**Table 1:** Information about *Cervus nippon* samples.

Population Name	Subspecies	Type	Region *	Count
DB (213)	*C. n. hortulorum*	Domesticated	Dongfeng, Jilin	35
			Changchun, Jilin	31
			Xingkai Lake, Heilongjiang	35
			Siping, Jilin	31
			Shuangyang, Jilin	33
			Dunhua, Jilin	17
			Tonghua, Jilin	30
DB (5)	*C. n. hortulorum*	Wild	Ussuriysk	5
HN (4)	*C. n. kopschi*	Wild	Qingliangfeng, Zhejiang	4
SC (9)	*C. n. sichuanicus*	Wild	Tiebu, Sichuan	9
TW (4)	*C. n. taiouanus*	Wild	Taipei, Taiwan	2
			Weihai, Shandong	2
BH (7)	*C. n. yesoensis*	Wild	Ashoro, Hokkaido	7
BZ (29)	*C. n. centralis*	Wild	Goyozan, Honshu	6
			Shimane, Honshu	5
			Yamaguchi, Honshu	9
			Tsushima, Tsushima	5
			Nikko, Honshu	4
WJ (11)	*C. n. yakushimae*	Wild	Tanegashima, Tanegashima	3
			Yakushima, Yakushima	6
			Miyanoura, Yakushima	1
			Yoshida, Yakushima	1
ZM (21)	*C. n. nippon*	Wild	Miyazaki, Kyushu	10
			Sata, Kagoshima/Kyushu	3
			Nagasaki, Kyushu	8

* All the domestic samples have been registered and stored in the Special Animal Resources library.

**Table 2:** Genetic diversity in eight populations of sika deer, based on 13 PCGs.

Population	Size *	Count	Hd (±SE)	π (±SE)
DB	217	38	0.700 (±0.015)	0.00624 (±0.0002)
HN	4	3	0.833 (±0.111)	0.00022 (±4 × 10^−5^)
SC	5	4	0.900 (±0.072)	0.00063 (±7.60 × 10^−5^)
TW	4	4	1.000 (±0.089)	0.00053 (±7.5 × 10^−5^)
BH	5	2	0.400 (±0.106)	0.00014 (±3.58 × 10^−5^)
BZ	25	5	0.793 (±0.009)	0.01018 (±0.0004)
WJ	9	6	0.889 (±0.030)	0.00636 (±0.0004)
ZM	21	10	0.910 (±0.008)	0.00329 (±5.02 × 10^−5^)
Total	290	72	0.917 (±0.000)	0.01430 (±4.58 × 10^−5^)

* Size: number of individuals; count: number of haplotypes.

**Table 3:** The number of subspecies-specific SNPs in 13 PCGs.

Population	DB	HN	SC	TW	BH	BZ	WJ	ZM
NAD1	0	2	8	8	2	0	2	0
NAD2	0	0	0	0	0	0	0	0
COX1	0	2	10	4	0	0	1	0
COX2	0	0	0	2	0	0	0	0
ATP8	0	3	0	3	0	0	0	0
ATP6	0	1	5	1	1	0	1	0
COX3	0	3	2	3	0	0	2	0
NAD3	0	0	1	1	0	0	0	0
NAD4L	0	0	0	0	0	0	0	0
NAD4	0	7	2	4	1	0	3	0
NAD5	1	4	1	5	0	0	2	0
NAD6	0	2	2	2	1	0	2	0
CYTB	0	4	5	8	0	0	1	0
Total	1	28	36	40	5	0	14	0

**Table 4:** Pairwise estimates of genetic distance for eight populations, based on 13 PCGs.

Populations	DB	HN	SC	TW	BH	BZ	WJ	ZM
DB								
HN	0.00874							
SC	0.01254	0.01187						
TW	0.01353	0.01271	0.01032					
BH	0.03012	0.03074	0.03065	0.03199				
BZ	0.02983	0.02945	0.03055	0.03103	0.01984			
WJ	0.01254	0.03205	0.03316	0.03381	0.02904	0.01354		
ZM	0.03256	0.02916	0.03093	0.03119	0.02637	0.00953	0.00917	

**Table 5:** Estimated genetic diversity indicators of Y chromosome genes in eight populations of sika deer.

	DB	HN	SC	TW	BH	BZ	WJ	ZM
DB								
HN	0.00041							
SC	0.00020	0.00020						
TW	0.00041	0.00041	0.00020					
BH	0.00183	0.00183	0.00162	0.00142				
BZ	0.00189	0.00188	0.00168	0.00148	0.00007			
WJ	0.00191	0.00185	0.00164	0.00144	0.00005	0.00011		
ZM	0.00193	0.00186	0.00166	0.00145	0.00010	0.00017	0.000015	
Hd *	0.728	0.000	0.000	0.000	0.000	0.345	0.600	0.667
π	0.00038	0.00000	0.00000	0.00000	0.00000	0.00007	0.00012	0.00014

* Summery of genetic diversity metrics per population was shown in Table S7.

## Data Availability

The raw sequence data and analysis scripts in this study are available on reasonable request from the corresponding author.

## Supplementary Materials

The following supporting information can be downloaded at: https://www.mdpi.com/article/10.3390/ani15203022/s1. Figure S1: Pairwise Fst values between the eight populations; Figure S2: Phylogenetic tree and haplotype network diagram based on 13 PCGs; Figure S3: Sankey plot of mitochondrial and Y chromosome haplotype distribution; Table S1: Information of collected specimens in this study; Table S2: Primers used for the PCR amplifications in this study; Table S3: The pattern of TR units in the D-loop region of mitochondrial sequences; Table S4: The arrangement types of the TR units in all samples; Table S5: Frequency distribution of M haplotypes in eight populations of *Cervus nippon*; Table S6: AMOVA analysis on 13 PCGs in eight populations of sika deer; Table S7: Summery of genetic diversity metrics per population.

## Abbreviations

The following abbreviations are used in this manuscript:

CR	Control Region
DAPC	Discriminant Analysis of Principal Components
NCBI	National Center for Biotechnology Information
PCG	Protein-Coding Gene
PCR	Polymerase Chain Reaction
SNP	Single Nucleotide Polymorphism
TR	Tandem Repeat
TRU	Tandem Repeat Unit
PIC	Polymorphism Information Content

## References

[1] Harris R.B. (2015). Cervus nippon. The IUCN Red List of Threatened Species.

[2] Ohtaishi N., Gao Y. (1990). A review of the distribution of all species of deer (Tragulidae, Moschidae and Cervidae) in China. Mammal Rev..

[3] Nagata J., Masuda R., Kaji K., Kaneko M., Yoshida M.C. (1998). Genetic variation and population structure of the Japanese sika deer (*Cervus nippon*) in Hokkaido Island, based on mitochondrial D-loop sequences. Mol. Ecol..

[4] van Doormaal N., Ohashi H., Koike S., Kaji K. (2015). Influence of human activities on the activity patterns of Japanese sika deer (*Cervus nippon*) and wild boar (*Sus scrofa*) in Central Japan. Eur. J. Wildl. Res..

[5] Cao Z., Wang D., Cui Y., Huang F., Liu Y., Dai J., Wu W., Dai Z., Xie J., Zhu X. (2025). Diet, nutrient characteristics and gut microbiome between summer and winter drive adaptive strategies of East China sika deer (*Cervus nippon* kopschi) in the Yangtze River basin. BMC Microbiol..

[6] Liu H., Zhu B., Wang T., Dong Y., Ju Y., Li Y., Su W., Zhang R., Dong S., Wang H. (2025). Population genomics of sika deer reveals recent speciation and genetic selective signatures during evolution and domestication. BMC Genom..

[7] Takatsuki S. (2009). Effects of sika deer on vegetation in Japan: A review. Biol. Conserv..

[8] Tsukada H., Ishikawa K., Shimizu N. (2012). Damage to round bale silage caused by sika deer (*Cervus nippon*) in central Japan. Grassl. Sci..

[9] Li H. (2003). Study on the performance of the velvet antler of Sika Deer). Chin. J. Anim. Sci..

[10] Tamate H.B., Tatsuzawa S., Suda K., Izawa M., Doi T., Sunagawa K., Miyahira F., Tado H. (1998). Mitochondrial DNA Variations in Local Populations of the Japanese Sika Deer *Cervus nippon*. J. Mammal..

[11] Lyu X.P., Wei F.W., Li M. (2006). Genetic diversity of Chinese sika deer (*Cervus nippon*) and its systematic relationship with Japanese sika deer). Chin. Sci. Bull..

[12] Wu H., Wan Q.H., Fang S.G. (2004). Two genetically distinct units of the Chinese sika deer (*Cervus nippon*): Analyses of mitochondrial DNA variation. Biol. Conserv..

[13] Nagata J., Masuda R., Tamate H.B., Hamasaki S.-I., Ochiai K., Asada M., Tatsuzawa S., Suda K., Tado H., Yoshida M.C. (1999). Two genetically distinct lineages of the sika deer, *Cervus nippon*, in Japanese islands: Comparison of mitochondrial D-loop region sequences. Mol. Phylogenet. Evol..

[14] Nagata J. (2009). Two Genetically Distinct Lineages of the Japanese Sika Deer Based on Mitochondrial Control Regions.

[15] Ba H., Wu L., Liu Z., Li C. (2014). An examination of the origin and evolution of additional tandem repeats in the mitochondrial DNA control region of Japanese sika deer (*Cervus nippon*). Mitochondrial DNA.

[16] Tanaka K., Hoshi A., Nojima R., Suzuki K., Takiguchi H., Takatsuki S., Takizawa T., Hosoi E., Tamate H.B., Hayashida M. (2020). Genetic Variation in Y-Chromosome Genes of Sika Deer (*Cervus nippon*) in Japan. Zool. Sci..

[17] Pereira F., Queirós S., Gusmão L., Nijman I.J., Cuppen E., Lenstra J.A., Consortium E., Davis S.J., Nejmeddine F., Amorim A. (2009). Tracing the history of goat pastoralism: New clues from mitochondrial and Y chromosome DNA in North Africa. Mol. Biol. Evol..

[18] Ramírez O., Ojeda A., Tomàs A., Gallardo D., Huang L., Folch J., Clop A., Sanchez A., Badaoui B., Hanotte O. (2009). Integrating Y-chromosome, mitochondrial, and autosomal data to analyze the origin of pig breeds. Mol. Biol. Evol..

[19] Bidon T., Janke A., Fain S.R., Eiken H.G., Hagen S.B., Saarma U., Hallström B.M., LeComte N., Hailer F. (2014). Brown and polar bear Y chromosomes reveal extensive male-biased gene flow within brother lineages. Mol. Biol. Evol..

[20] Dong Y., Li Y., Wang T., Liu H., Zhang R., Ju Y., Su W., Tamate H., Xing X. (2022). Complete mitochondrial genome and phylogenetic analysis of eight sika deer subspecies in northeast Asia. J. Genet..

[21] Smith J., Lee A. (2021). Sequence analysis using DNAMAN. Bioinformatics.

[22] Kumar S., Stecher G., Suleski M., Sanderford M., Sharma S., Tamura K. (2024). MEGA12: Molecular Evolutionary Genetic Analysis Version 12 for Adaptive and Green Computing. Mol. Biol. Evol..

[23] Vaidya G., Lohman D.J., Meier R. (2011). SequenceMatrix: Concatenation software for the fast assembly of multi-gene datasets with character set and codon information. Cladistics.

[24] Rozas J., Ferrer-Mata A., Sánchez-DelBarrio J.C., Guirao-Rico S., Librado P., Ramos-Onsins S.E., Sánchez-Gracia A. (2017). DnaSP 6: DNA Sequence Polymorphism Analysis of Large Data Sets. Mol. Biol. Evol..

[25] PopArt. http://popart.otago.ac.nz.

[26] Clement M., Snell Q., Walker P., Posada D., Crandall K.A. (2002). TCS: Estimating gene genealogies. Parallel Distrib. Process. Symp. Int. Proc..

[27] Excoffier L., Lischer H.E.L. (2010). Arlequin suite ver 3.5: A new series of programs to perform population genetics analyses under Linux and Windows. Mol. Ecol. Resour..

[28] Benson G. (1999). Tandem repeats finder: A program to analyze DNA sequences. Nucleic Acids Res..

[29] Jombart T. (2008). adegenet: A R package for the multivariate analysis of genetic markers. Bioinformatics.

[30] Mao Y., Sun X., Shen J., Gao F., Qiu G., Wang T., Nie X., Zhang W., Gao Y., Bai Y. (2019). Molecular Evolutionary Analysis of Potato Virus Y Infecting Potato Based on the VPg Gene. Front. Microbiol..

[31] Štohlová Putnová L., Štohl R., Ernst M., Svobodová K. (2021). A Microsatellite Genotyping-Based Genetic Study of Interspecific Hybridization between the Red and Sika Deer in the Western Czech Republic. Animals.

[32] Kalb D.M., Delaney D.A., DeYoung R.W., Bowman J.L. (2019). Genetic diversity and demographic history of introduced sika deer on the Delmarva Peninsula. Ecol. Evol..

[33] Zhou Y.N. (2018). Analysis of Maternal and Paternal Types of Stud Sika Deer Based on Mitochondrial DNA and Y Chromosome Gene Fragments. Master’s Thesis.

[34] Borowski Z., Świsłocka M., Matosiuk M., Mirski P., Krysiuk K., Czajkowska M., Borkowska A., Ratkiewicz M. (2016). Purifying Selection, Density Blocking and Unnoticed Mitochondrial DNA Diversity in the Red Deer, *Cervus elaphus*. PLoS ONE.

[35] Nabata D., Masuda R., Takahashi O. (2004). Bottleneck effects on the sika deer *Cervus nippon* population in Hokkaido, revealed by ancient DNA analysis. Zool. Sci..

[36] Goodman S.J., Tamate H.B., Wilson R., Nagata J., Tatsuzawa S., Swanson G.M., Pemberton J.M., McCullough D.R. (2001). Bottlenecks, drift and differentiation: The population structure and demographic history of sika deer (*Cervus nippon*) in the Japanese archipelago. Mol. Ecol..

[37] Petr M., Hajdinjak M., Fu Q., Essel E., Rougier H., Crevecoeur I., Semal P., Golovanova L.V., Doronichev V.B., Lalueza-Fox C. (2020). The evolutionary history of Neanderthal and Denisovan Y chromosomes. Science.

[38] Liu S.Q. (2017). Study on the Origin of Chinese Domestic Horses Based on Genetic Variations of Y-Chromosome. Ph.D. Thesis.

