# Supplementary figures and images for: Genetic Diversity and Population Structure of Sika Deer (Cervus nippon) Inferred by mtDNA and Y-Chromosomal Genes
Source: Animals (Basel). 2025 Oct 17;15(20):3022. doi: 10.3390/ani15203022 (PMC12561076; doi:10.3390/ani15203022)

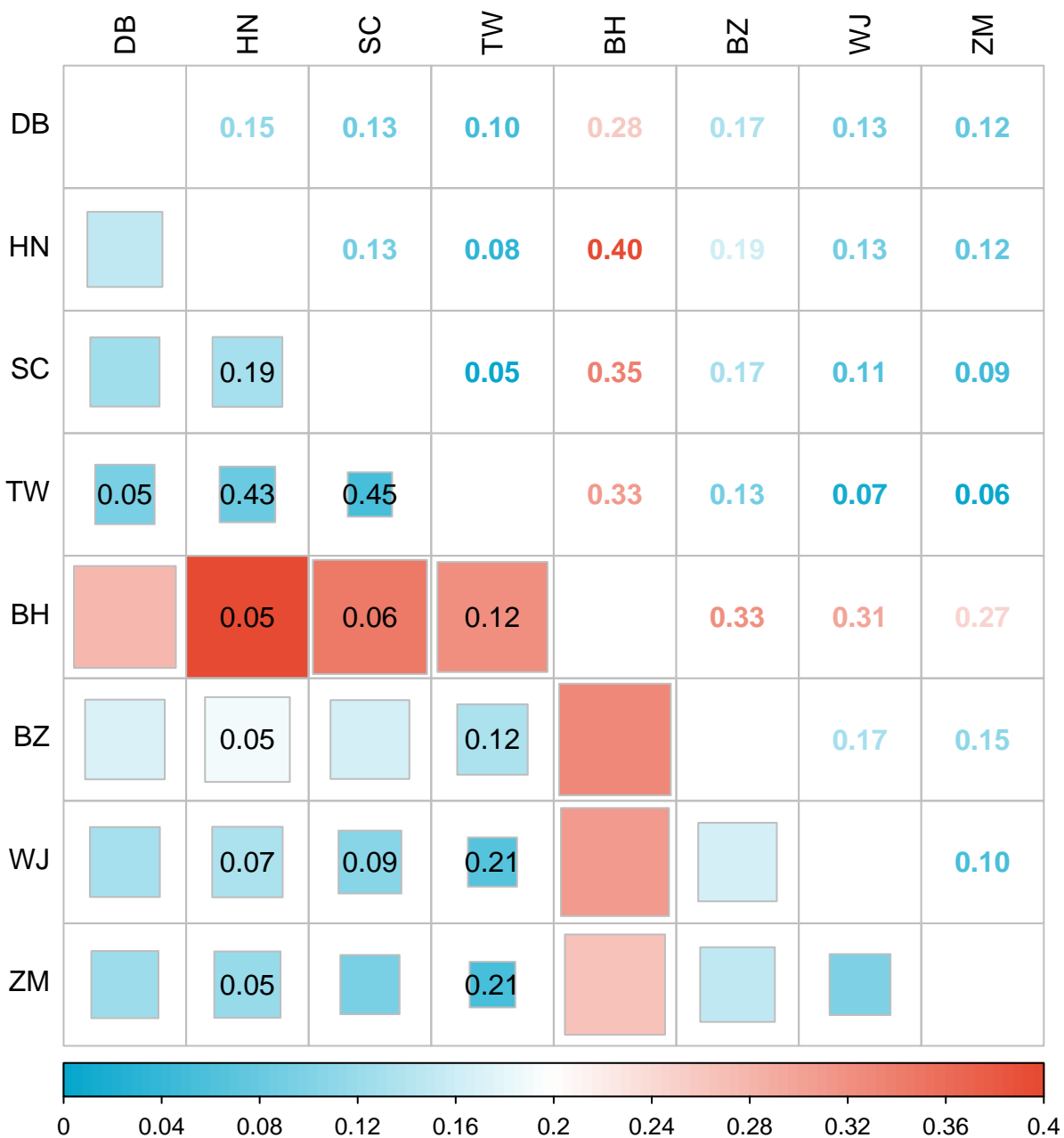

Supplement: Supplementary file 1 [file animals-15-03022-s001.zip › Figure S1.pdf]

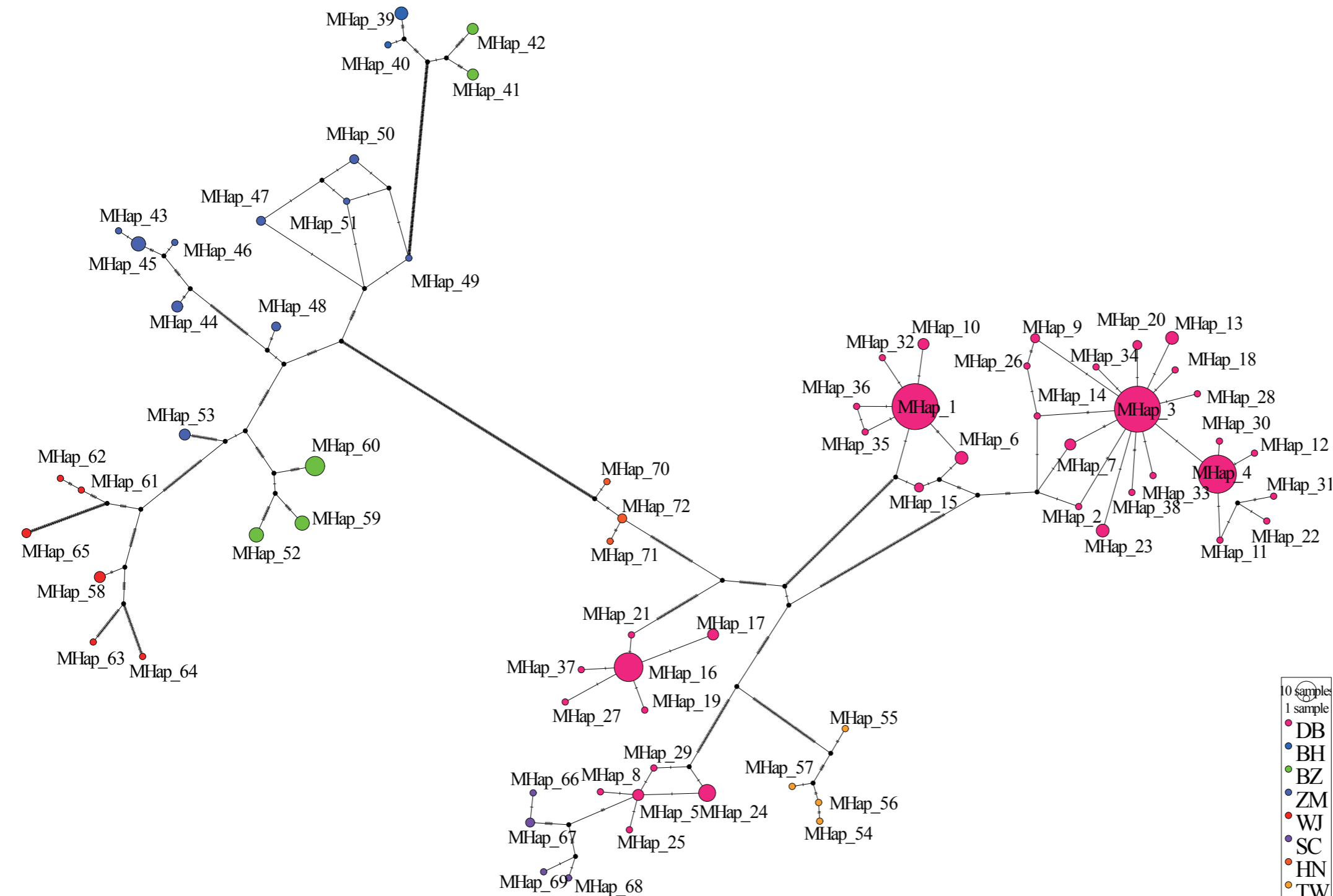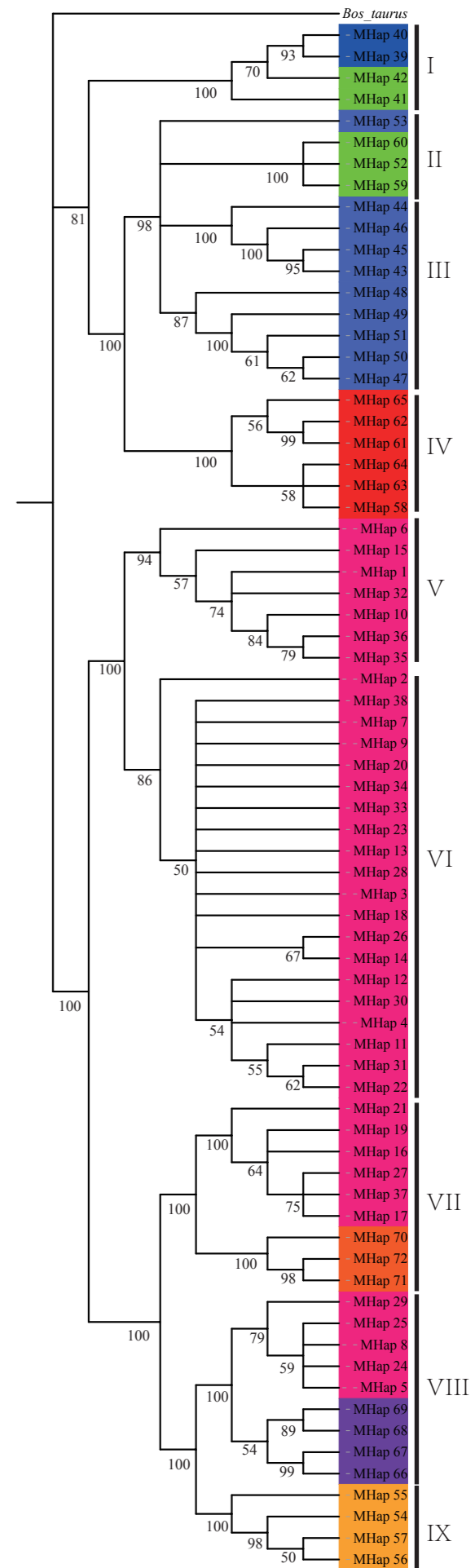

Supplement: Supplementary file 1 [file animals-15-03022-s001.zip › Figure S2.pdf]

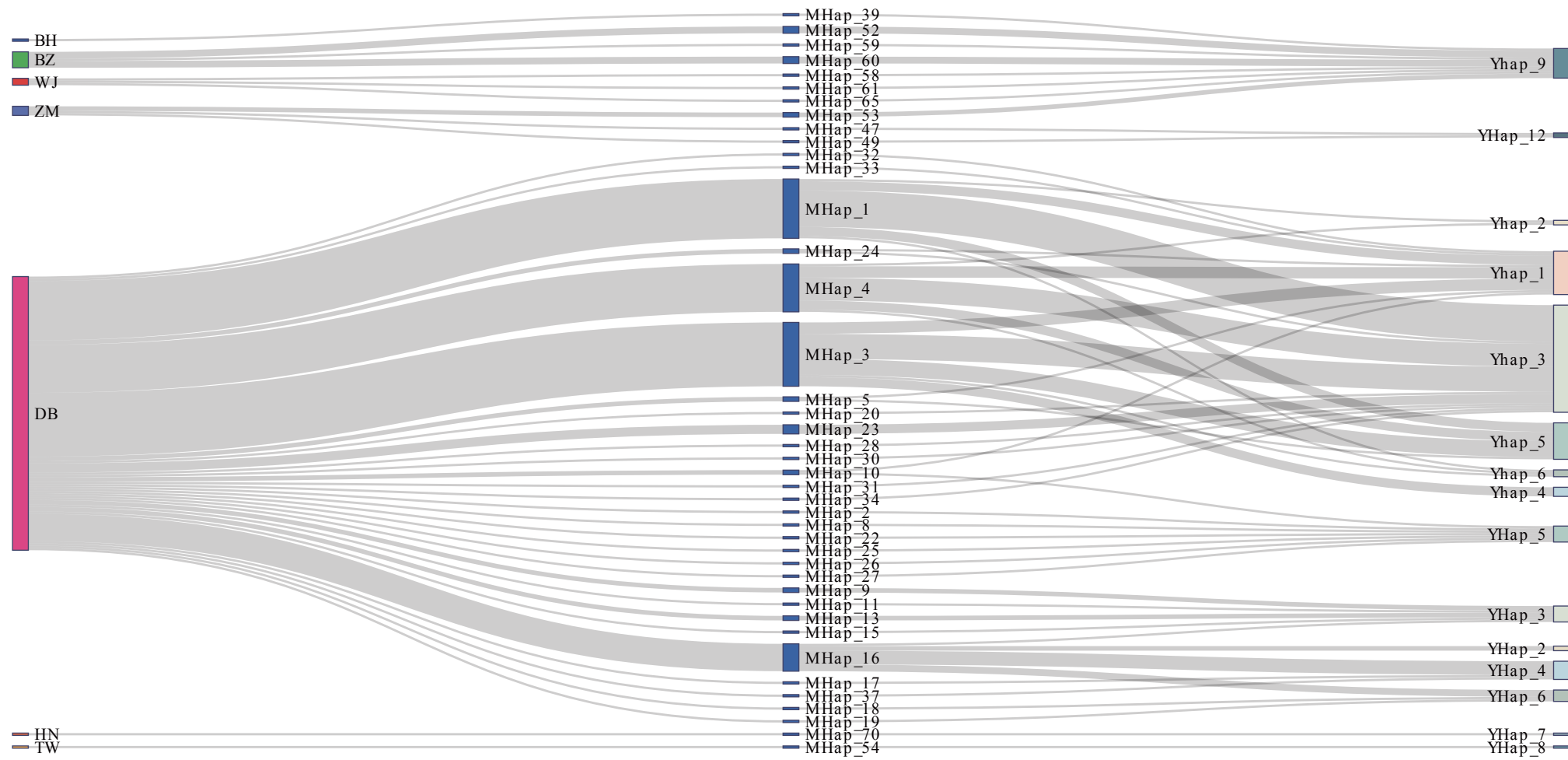

Supplement: Supplementary file 1 [file animals-15-03022-s001.zip › Figure S3.pdf]
